# The National Institutes of Health Microphysiological Systems Program focuses on a critical challenge in the drug discovery pipeline

**DOI:** 10.1186/scrt361

**Published:** 2013-12-20

**Authors:** Margaret L Sutherland, Kristin M Fabre, Danilo A Tagle

**Affiliations:** 1 National Institute of Neurological Disorders and Stroke, National Institutes of Health, 6001 Executive Blvd., Bethesda, MD 20892, USA; 2 National Center for Advancing Translational Sciences, National Institutes of Health, 6701 Democracy Blvd., Bethesda MD 20892, USA

## Abstract

The National Institutes of Health has partnered with the US Food and Drug Administration and the Defense Advanced Research Projects Agency to accelerate the development of human microphysiological systems (MPS) that address challenges faced in predictive toxicity assessment and efficacy analysis of new molecular entities during the preclinical phase of drug development. Use of human MPS could provide better models for predicting the efficacy of new molecular entities in clinical trials. It is also anticipated that improvements in predicting drug toxicities early in the drug development process through the use of MPS or human organs-on-a-chip will decrease the need to withdraw new therapies from the market and minimize or eliminate deaths due to unidentified drug toxicities.

## Challenges in moving promising new drugs to the marketplace

Although advances in basic and preclinical science continue to fuel the drug discovery pipeline, only a small fraction of compounds in the pipeline meet criteria for approval by the US Food and Drug Administration (FDA) [1]. Current success rates for compounds that reach phase II and phase III clinical trials have dropped to 18% and 50%, respectively [2,3], with failures in the clinic and removal prior to FDA review being attributed to a lack of efficacy, unidentified toxicities in the preclinical phase, and an ever-changing business strategy for some of the drug developers [2,3]. Since the drug discovery pipeline is both labor and resource intensive, alternative approaches that would enable early indications and potentially more reliable readouts of toxicity or efficacy could provide significant improvements in the success rate and associated cost benefits for this process. To address this challenge in drug development and regulatory science, the National Center for Advancing Translational Sciences (NCATS), through the Cures Acceleration Network and in conjunction with the National Institutes of Health (NIH) Common Fund, has invested $70 million over a 5-year period to launch the Microphysiological Systems (MPS) Program or Organs-on-Chips Program.

## The NIH Microphysiological Systems Program targets novel human cell-based systems for predictive drug toxicity

The NIH MPS Program is part of a coordinated effort between the NIH, the Defense Advanced Research Projects Agency (DARPA), and the FDA to accelerate the development of human MPS that will improve the reliability to identify human drug toxicities and predict the potential efficacy of a drug in a human population prior to use of the drug in late-stage clinical studies. While the NIH and DARPA independently fund and manage their own MPS programs, coordination of these programs occurs through biannual meetings with NIH and DARPA investigators. These meetings provide opportunities not only to share advances in platform development, cell source characterization, but also to establish collaborations that will help accelerate the development, validation and integration of robust human MPS.

The goal of the NIH MPS Program is to create and integrate MPS that utilize human primary or stem cell sources, which are sustainable over a 4-week period and functionally represent the 10 major organ systems: circulatory, respiratory, integumentary, reproductive, endocrine, gastrointestinal, nervous, urinary, musculoskeletal, and immune. The 19 investigators funded under the MPS Program are supported through 2-year (Table 1) and 5-year (Table 2) cooperative agreements, where annual milestones determine the successful progression of each project. The MPS cooperative agreement mechanism also facilitates monthly reviews of progress on individual projects where scientific program staff, representing 15 NIH institutes, provide advice on system metrics and assist in the identification of available resources that can help achieve the aggressive timelines of the program. To further serve the objectives of the cooperative agreement, the MPS Program has implemented a communications interface sponsored by NCATS, which is design to facilitate information exchange and the development of MPS organ and cell source standards among the investigators.

**Table 1:** The U18: 2-year funded National Institutes of Health Microphysiological Systems Program investigators

Primary U18 investigator	Institution	Title
James M Wells, PhD	Cincinnati Children's Hospital Medical Center, OH	Generating human intestinal organoids with an enteric nervous system
Angela Christiano, PhD	Columbia University Health Sciences, New York City, NY	Modeling complex disease using induced pluripotent stem cell-derived skin constructs
Mark Donowitz, MD	Johns Hopkins University, Baltimore, MD	Human intestinal organoids: preclinical models of noninflammatory diarrhea
Thomas Hartung, MD, PhD	Johns Hopkins University, Baltimore, MD	Three-dimensional model of human brain development for studying gene/environment interactions
John P Lynch, MD, PhD	University of Pennsylvania, Philadelphia, PA	Modeling oxidative stress and DNA damage using a gastrointestinal organotypic culture system
Rocky S Tuan, PhD	University of Pittsburgh, PA	Three-dimensional osteochondral micro-tissue to model pathogenesis of osteoarthritis
Joan E Nichols, PhD	The University of Texas Medical Branch at Galveston, TX	Three-dimensional human lung model to study lung disease and formation of fibrosis

The emphasis of these 2-year grants is the incorporation of human embryonic, induced pluripotent stem cell and progenitor cell sources into organ-specific microphysiological systems. The multicellular models developed will represent the three-dimensional architecture and cellular composition of the organ being modeled, and will lead to the development of microphysiological systems that represent the function of the target organ under normal physiological conditions and disease states.

**Table 2:** The UH2/UH3: 5-year funded National Institutes of Health Microphysiological Systems Program investigators

Primary UH2 investigators	Institution	Title
Gordana Vunjak-Novakovic, PhD	Columbia University in the City of New York, NY	Integrated heart-liver-vascular systems for drug testing in human health and disease
Michael L Shuler, PhD and James J Hickman, PhD	Cornell University, Ithaca, NY and University of Central Florida, FL	Microphysiological systems and low-cost microfluidic platform with analytics
George A Truskey, PhD	Duke University, Durham, NC	Circulatory system and integrated muscle tissue for drug and tissue toxicity
Kevin K Parker, PhD	Harvard University, Cambridge, MA	Human cardiopulmonary system on a chip
Linda G Griffith, PhD	Massachusetts Institute of Technology, Cambridge, MA	All-human microphysical model of metastasis and therapy
James A Thomson, VMD, PhD	Morgridge Institute for Research at the University of Wisconsin-Madison, WI	Human induced pluripotent stem cell and embryonic stem cell-based models for predictive neural toxicity and teratogenicity
Teresa K Woodruff, PhD	Northwestern University, Chicago, IL	*Ex vivo *female reproductive tract integration in a three-dimensional microphysiologic system
Kevin E Healy, PhD and Luke P Lee, PhD	University of California, Berkeley, CA	Disease-specific integrated microphysiological human tissue models
Steven C George, MD, PhD	University of California, Irvine, CA	An integrated *in vitro *model of perfused tumor and cardiac tissue
D Lansing Taylor, PhD and Martin L Yarmush, MD, PhD	University of Pittsburgh, PA and Rutgers University, NJ	Three-dimensional biomimetic liver sinusoid construct for predicting physiology and toxicity
Jonathan Himmelfarb, MD	University of Washington, Seattle, WA	A tissue-engineered human kidney microphysiological system
John P Wikswo, PhD, Damir Janigro, PhD, Donna Webb, PhD and Kevin Niswender, PhD	Vanderbilt University, Nashville, TN	Neurovascular unit on a chip: chemical communication, drug and toxin responses

The National Institutes of Health has issued 12 (UH2/UH3) 5-year awards to fund the development of microphysiological system platforms that represent a number of human organ systems. In the first 2-year phase of the Microphysiological Systems (MPS) Program (UH2), investigators will create functionally and physiologically relevant microsystems, which will accurately reflect tissue complexity, and genomic diversity. The scientific objectives of the UH2 development phase include demonstration that the microphysiological system: represents the multicellular architecture of the target organ; has viable and reproducible function for 4 weeks under physiological conditions; accurately recapitulates disease and pathogenic phenotypes; and can be adapted to high content screening for repeated dose efficacy testing and toxicology. The MPS investigators who successfully achieve the UH2 milestone goals will advance to the UH3 (3-year) phase, which involves MPS integration onto a common platform, and validation of the accurate function of integrated systems on the platform through blinded testing of drugs with known toxicities and mechanisms of action.

The concept of developing standards around the microphysiological organ systems under development by NIH investigators is driven by the need to provide a MPS consensus view on the minimal functional requirements of a system to accurately mimic the function of the human organ. A similar approach is being applied to the induced pluripotent stem cell sources being utilized in MPS development, wherein any induced pluripotent stem cell source must: demonstrate a normal karyotype; be free of bacterial and mycoplasma contamination; demonstrate expression of stem cell markers; demonstrate successful silencing of transgenes used in the reprogramming phase; and have the ability to differentiate along mesoderm, ectoderm and endoderm germ layer lineages. Through the development of MPS and cell source standards, collaborations and information exchange, it is anticipated that within the next 5 years the collective efforts of the NIH grantees will culminate in MPS that will provide improved research tools for efficacy and toxicology screening of new molecular entities.

To evaluate the potential improvement MPS provide over traditional *in vivo *animal toxicology, a set of training compounds has been selected based on known toxicities in the human population. This training set of compounds addresses organ-specific toxicities that will be tested across the various MPS platforms. A second set of drugs, where the identity is blinded to the investigators, will be used for validation to ensure an unbiased evaluation of the reliability and predictability of these systems. The global application of MPS in new drug toxicity and efficacy testing, and recognition of these systems by regulatory agencies such as the FDA, will require coordination with standards agencies like the International Conference on Harmonization. The International Conference on Harmonization has developed a comprehensive set of safety guidelines to uncover potential risks such as carcinogenicity, genotoxicity and reprotoxicity, and these safety guidelines have been adopted by international regulatory agencies.

## Defense Advanced Research Projects Agency and US Food and Drug Administration

To provide advanced engineering and platform development that will support the integration of 10 human organ systems onto a single platform with a biofluidics infrastructure that can link the various organ systems, a parallel and complementary effort to the NIH MPS Program is funded by DARPA. Dr Linda Griffith (Massachusetts Institute of Technology, MA) and Dr Don Ingber (Wyss Institute, Harvard University, MA) are DARPA investigators tasked with the stepwise integration and validation of 10 human organ systems onto a shared platform that supports the viability of the integrated systems for a 4-week period. Like the NIH MPS Program, the DARPA Program represents a 5-year, $75 million commitment.

As defined in a memorandum of understanding between the NIH and the FDA, for the NIH MPS Program the FDA provides key guidance for development of robust compound libraries and physiological readouts that reflect toxicities both within a single organ system and across multiple organ systems.

## Why animal models can fail

The challenge of accurately predicting drug toxicities and efficacies is in part due to inherent species differences in drug metabolizing enzyme activities [4,5] and cell type-specific sensitivities to toxicants [5]. For example, a dramatic difference between rat and human drug metabolism is demonstrated by the metabolism of coumarin. Species differences in coumarin toxicity appear to be mediated through two major phase I metabolic pathways. The first involves the conversion of coumarin by cytochrome P450 CYP2A enzymes to the nontoxic metabolite 7-hydroxycoumarin. Whereas in humans this reaction is very efficient, in rats CYP2A enzymes preferentially catalyze 7α-hydroxylation of testosterone rather than coumarin 7-hydroxylation [6] and therefore the formation of 7-hydroxycoumarin is extremely low in rats and the lack of 7-hydroxycoumarin is thought to render rats more susceptible to hepatotoxicity [7]. The second pathway involves detoxification of the epoxide intermediate coumarin 3,4-epoxide. In humans, coumarin 3,4-epoxide spontaneously rearranges to *o*-hydroxyphenylacetaldehyde, which is further detoxified by oxidation to *o-*hydroxyphenylacetic acid. In rats, this conversion to *o-*hydroxyphenylacetic acid is 50 times lower than in humans, and represents a second major species difference in coumarin-mediated heptotoxicity [8]. Certainly, a clear understanding of the role of drug metabolism in toxicity and potential species differences in response to toxic metabolites will aid in the development of safer drugs.

Inherent differences in cell type-specific sensitivities between species may also be an important differentiating factor for the prediction of drug toxicities. An example of differential cell toxicities across species is observed with bizelesin, a potent synthetic derivative of the anticancer agent CC-1065 that preferentially alkylates and binds the minor groove of DNA. Results with myelopoietic cells *in vitro*, in the absence of liver metabolism, reproduced *in vivo *species differences in myelosuppression, hence showing that murine cells are 1,000-fold more sensitive than human or canine cells [9]. In the case of the antidiabetic drug troglitazone, metabolism by CYP3A4 and CYP3a1 in human and rat, respectively, results in a reactive quinone intermediate that can bind to proteins and nucleic acids and can potentially produce oxidative stress through the generation of reactive oxygen species via redox cycling or depletion of the oxidative stress protective tripeptide, glutathione. Using rat and human hepatocyte monolayer cultures, Lauer and colleagues demonstrated strong species differences and marked alterations in expressions of genes involved in xenobiotic metabolism as well as oxidative stress after treatment with troglitazone [6]. The higher sensitivity of human hepatocytes towards troglitazone treatment in contrast to the weaker effects observed in rat hepatocytes at least partially explains why the strong hepatotoxic effects of troglitazone in the human population could not be predicted from regulatory animal studies [6]. The results of these *in vitro *studies suggest that in addition to differences in drug metabolism, target cell sensitivity differences may also account for species differences in toxicity.

A review of FDA-approved drugs released from 1975 to 1999 estimated that 2.9% of these marketed drugs were withdrawn from the market due to severe adverse drug effects [10]. According to Temple and Himmel, hepatotoxicity was the most frequent single reason for removing drugs from the market during this timeframe, and probably will still be the most important single toxicity leading to withdrawal or significant modification of labeling for FDA-approved drugs going forward [11]. Examples of drugs removed from the market due to hepatoxicity during this time period include ticrynafen, benoxaprofen, bromfenac, and troglitazone. In addition to hepatoxicity, six drugs were withdrawn from the market because of hemolytic anemia (nomifensine maleate), hemolytic anemia, renal and hepatic injury (temafloxacin), thromboembolism (azaribine), anaphylaxis (zomepirac sodium), acute but reversible renal failure (suprofen), and increased mortality (flosequinan). Based on estimates from Lasser and colleagues, the probability of a new drug acquiring black box warnings or being withdrawn from the market over 25 years is approximately 20% [12]. Through development of human cell-based MPS, it is hoped that toxicities as mentioned above will be detected earlier, preferably during the preclinical phase of drug development.

Given the list of FDA drugs withdrawn due to unpredicted toxicities in the human population, a key goal of the NIH MPS Program is to accurately evaluate the predictive value of these systems in determining drug toxicity and efficacy. This goal can be achieved through either development of a single microphysiological system, such as a liver organoid system that can reliably predict human hepatoxicity, or through a more complex platform where multiple MPS predict multiorgan toxicities to a given challenge drug. The multisystem platform approach will also assist in understanding the time dependence and magnitude of drug-drug interactions; for instance, how one drug may increase the blood concentration of another, as seen in the effect of mibefradil on midazolam blood concentrations [12]. Understanding the limitations of these model systems will also be important. For instance, in cases where recognition of toxicity in the human population has been delayed - such as seen in the marked hypo tension with clozapine, the pulmonary fibrosis and marrow toxicity with tocainide, the valvulopathy associated with fenfluramine hydrochloride and the subarachnoid hemorrhage associated with phenyl propanol-amine [13] - the application of relatively short-term exposures of drugs in the MPS may limit the predictive value of the system for delayed toxicities.

## National Center for Advancing Translational Sciences and the Microphysiological Systems Program

The MPS Program is an excellent flagship program for NCATS, established in 2011 with a mission to catalyze the generation of innovative methods and technologies that will enhance the development, testing, and implementation of diagnostics and therapeutics across a wide range of diseases and conditions. The opportunities for significant advancements in the prediction of human drug toxicities through the development of MPS requires a multidisciplinary approach that relies on an understanding of human physiology, stem cell biology, material sciences and bioengineering. At the end of the 5-year funding period it is anticipated that the availability of these systems to a broader research community will foster a multitude of new research applications including, but not limited to, studies in environment exposures, reproduction and development, infectious diseases, cancer, countermeasures for chemical warfare, immune responses and neuroinflammation.

## Abbreviations

CYP: cytochrome P450; DARPA: Defense Advanced Research Projects Agency; FDA: US Food and Drug Administration; MPS: microphysiological systems; NCATS: National Center for Advancing Translational Sciences; NIH: National Institutes of Health.

## Competing interests

The authors declare that they have no competing interests.

## Declarations

Publication of this supplement has not been supported by sponsorship. Articles have undergone the journal's standard review process. The Editors declare that they have no competing interests.

This article has been published as part of *Stem Cell Research & Therapy *Volume 4 Supplement 1, 2013: Stem cells on bioengineered microphysiological platforms for disease modeling and drug testing. The full contents of the supplement are available online at http://www.stemcellres.com/supplements/4/S1.
